# Seven-Parameter Polynomial Fits Better to the Moisture Sorption Isotherms of Oil-Type Peony Seeds and Cake

**DOI:** 10.3390/foods15081298

**Published:** 2026-04-09

**Authors:** Xingjun Li, Bing Dai, Chang Liu, Qingyan Shu

**Affiliations:** 1 Academy of National Food and Strategic Reserves Administration, National Engineering Research Center for Grain Storage and Transportation, Beijing 102209, China; bingdai3@126.com (B.D.); shlglc@126.com (C.L.); 2 College of Grain and Strategic Reserves, Henan University of Technology, Zhengzhou 450001, China; 3 State Key Laboratory of Plant Diversity and Specialty Crops, Institute of Botany, Chinese Academy of Sciences, Beijing 100093, China; shuqy@ibcas.ac.cn

**Keywords:** peony seed, adsorption and desorption isotherms, isosteric heat of sorption, monolayer moisture content, microstructure, online monitoring moisture content

## Abstract

As an emerging oilseed crop in China, peony seed oils account for 0.41% of the annual production of Chinese edible vegetable oils, and the oil-type peony seed is rich in alpha-linolenic acid (ALA). Moisture content and temperature are key factors in the storage of oilseeds. In this study, the adsorption and desorption isotherms of ten species of peony seeds and one species of cake were determined in the range of 20–30 °C and 10–90% equilibrium relative humidity (ERH). The adsorption and desorption isotherms of peony seeds and cake were type II (sigmoidal) or type III curves. Nine equilibrium moisture content (EMC) equations were used to fit the isotherms of peony samples, with the optimal equations being our developed 7-parameter polynomial (Poly), modified Halsey equation (MHAE), and modified Oswin equation (MOE). For Poly, the fitting parameter determination coefficient (R^2^) was 0.9816–0.9986, and the mean relative error (MRE) was 0.83–6.52%; for MHAE, R^2^ was 0.7815–0.9973, and MRE was 4.18–17.84%. Poly contains the terms of temperature and ERH interaction; therefore, Poly could analyze the safe moisture content of peony seeds and cake during storage and transportation, and the three-parameter reversible MHAE could be used for calculating the sorption isosteric heats. The adsorption monolayer moisture content (M_0_) in peony seeds and cake estimated by MGAB were 3.64 ± 0.42% and 4.28%, respectively, while their desorption M_0_ values, respectively, were 6.21 ± 0.47% and 4.83%. At ERH ≤ 65%, for preventing the growth of storage pests and fungi, the absolutely safe storage moisture content (MC) predicted by Poly at 25 °C and 65% ERH was 12.48% wet basis (w.b.) for seeds and 11.92% for cake. The heat of sorption of peony seeds and cake approached that of pure water at about 11% and 15% w.b. MC estimated by the MHAE model, respectively. Microstructure analysis showed that the rich liposomes in peony seeds were attached to the inner surface of the cell wall and the outer surface of the protein storage vacuole, and the rich protein bodies and hydrophilic polysaccharides explained why the safe storage moisture for yellow peony seeds was higher than for Ziyan Feishuang seeds. This study provides the basic data for drying simulation, and the safe storage and transportation of peony seed and cake products.

## 1. Introduction

*Paeonia suffruticasa* Andr. is a unique plant resource of China with a history of more than 2000 years of cultivation [1]. It has experienced three stages of utilization: medicinal, ornamental, and comprehensive. Oilseed peony is an important woody oil crop, and its leaves, flowers, seeds, oil and root bark can be eaten or used for medicinal purposes [2]. Peony seed oil is pressed from the seed kernels of “Fendan” peony (*Paeonia ostii*) and “Ziban” peony (*Paeonia rockii*) species, which belong to genus Paeonia in the family Paeoniaceae [3]. Peony seed oil, a new type of woody oil extracted from oilseed-type peony seeds, is rich in α-linolenic acid (ALA), linoleic acid, oleic acid and other unsaturated fatty acids (UFA), with total UFA content of more than 85%, of which the ALA content, respectively, is 5, 10, and 30 times that of walnut oil, soybean oil, and corn oil [4]. The ω-3 polyunsaturated fatty acid, ALA, is an indispensable nutrient for the human body, which plays an important role in maintaining the normal function of the nervous system, cardiovascular system, and visual system. In addition, a peony seed has the basic components of 31.22% crude fat, 20.35% crude protein, 9.9% starch, 7.82% soluble sugars, and 2.78% ash, and also contains flavonoids, fiber, pectin, and other substances [4]. A 6-to-8-year-old peony plant can produce 4500 to 6000 kg of peony seeds per hectare, which has large development potential [4]. In March 2011, the Chinese Ministry of Health approved the oil pressed from peony seeds as a new resource food, and the industry standard for Peony seed oil was issued in November 2014 [5]. The oilseed peony planting area in China is 13.4 million hectares, with an annual production of 0.126 million tonnes of edible oils and accounting for 0.41% annual production of edible vegetable oils [3]. Because a peony seed has high crude fat, the storage conditions—such as temperature and oxygen concentration—significantly influence lipid quality [3]. Lv et al. [6] suggested that the storage of ‘Fendan’ peony seeds in nylon mesh bags in bulk mode at room temperature, in vacuum packaging at room temperature, and in nylon mesh bags at 4 °C and at −20 °C, should not exceed 60, 150, 120, and 300 days, respectively, to prevent oil deterioration. To understand the processes of drying and ambient cooling of oil-type peony seeds, and their effects on water activity, and to improve physical control in storage, a sound knowledge of the relationship between the equilibrium moisture content (EMC) and equilibrium relative humidity (ERH) for peony seeds is essential.

In engineering and postharvest operations, equilibrium moisture isotherms are very informative for designing drying operation models and equipment, predicting the shelf life of food and grains, selecting suitable storage conditions, aeration cooling, and packaging materials, and calculating thermodynamic parameters such as enthalpy and entropy [7,8]. These operations directly affect product stability, energy efficiency, and quality preservation. A more explicit connection between the sorption data and their application in these unit processes would better position the study within the broader scope of food engineering and sustainable postharvest technologies [9,10]. The equilibrium moisture equations of staple cereals and oils have been evaluated in the past twenty years [11]. They are gradually being used for the smart design of different mechanical aeration processes, like cooling, re-moisturizing, and de-moisturizing aeration [12]. Despite the EMC/ERH data existing for other oil crops (e.g., soybean, sesame) [11], this study provides the first report of EMC/ERH data for peony seeds.

Because storage temperature and moisture content strongly influence enzymatic lipid degradation, insights from olive fruit blanching studies highlight the critical role of controlling water activity to preserve oil quality [13]. A peony seed has high contents of crude protein, starch, and soluble sugars [4], thus it was assumed in this study that hydrophilic proteins and polysaccharides adsorb water molecules [8,14]. Using peony cake with a small amount of oil as the control, this study will determine the desorption and adsorption equilibrium moisture isotherms of ten species of peony seeds, find optimal equations, determine safe storage moisture content (MC), and calculate isosteric heats, revealing the relationship of hydrophilic components to moisture adsorption, particularly how the hydrophilic structures of proteins and polysaccharides affect isothermal behavior. This study will provide guidance for drying design, safe storage management, transportation, and packaging of peony seeds and cake. Additionally, the influence of changes in storage conditions on quality parameters and the importance of optimizing storage conditions for preserving peony seed quality and extending shelf life were examined.

## 2. Materials and Methods

### 2.1. The Samples

**Figure 1:**
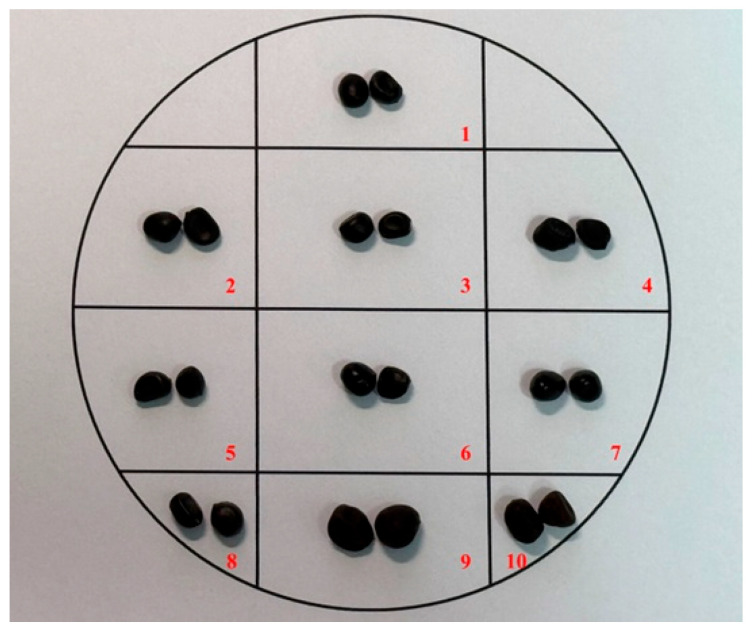
Photograph of ten peony seed samples. Notes: 1, a1; 2, a2; 3, a3; 4, a4; 5, a5; 6, a6; 7, a7; 8, a8; 9, a9; 10, a10.

**Figure 2:**
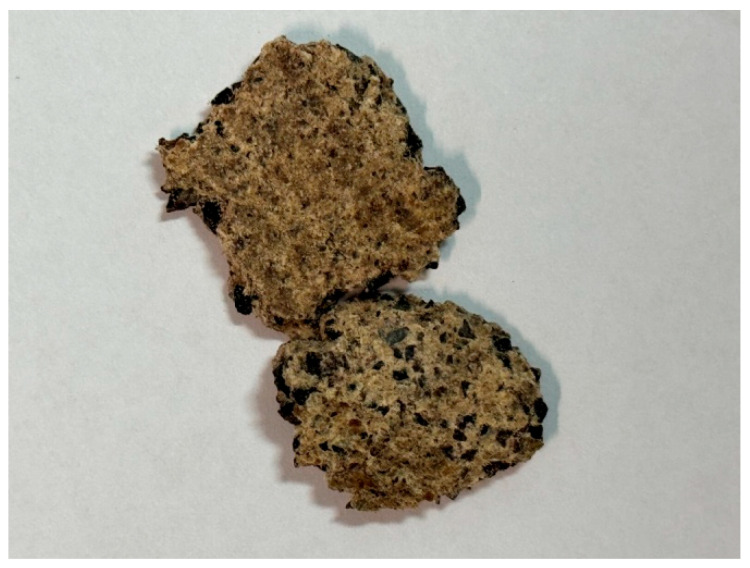
Photograph of Ziyun Mudan cake (a11).

Ten species of peony seeds with hulls and one species of cake were supplied by the Institute of Botany, Chinese Academy of Sciences, in 2024. The peony seeds and cake were separately stored in nylon mesh bags at 4 °C. Photographs and the initial moisture content are provided in Figure 1 and Figure 2 and Table 1, respectively. The overall seed shape (sub-globose, oval, or nearly round) and mature color (black or brownish-black) indicate the maturity of the peony seed. The peony cake was the cake formed after cold pressing peony kernels. The procedure for cold pressing peony seeds was as follows: Cleaning → Metering → Dehusking → Kernel/shell separation → Drying → Cold pressing → Filtration → Cold pressed oil and kernel cake [4]. The temperature during physical pressing was controlled below 60 °C.

The initial moisture content of the sample was determined by the oven dry method [15,16]. The samples (10 g) were ground in a crusher (JFSD-100, Jiading Grain and Oil Instrument Co., Ltd., Shanghai, China), and the moisture content in each powder was dried at 103 ± 0.5 °C in an oven (DHG-9070A, electric heating constant temperature blower drying oven, Blue Sky Chemical Instrument Factory, Hangzhou, China) for 3 h. The moisture content in this study was given in wet basis (w.b.). The sample shape was determined by human eyes.

### 2.2. Determination of Equilibrium Moisture Content

The moisture sorption isotherms of the 11 samples were determined using a dynamic moisture sorption device (SPS11-10μ, ProUmid Gmbh & Co. KG, Ulm, Germany). This integrated system can automatically gravimetrically determine the water vapor sorption of multiple samples in a test chamber under controlled temperature and equilibrium relative humidity (ERH) conditions. The moisture sorption analyzer has a temperature accuracy of ±0.1 K, and ERH accuracy of ±0.6% RH at 23 ± 5 °C in the range of 0–98% RH. A sample aluminum pan (No. 1) was unloaded and was used for drift compensation of the measured values, and No. 2–11 pans were loaded with peony samples. After determining the initial moisture content (IMC, % wet basis) of each samples, two internal parallel samples (each ca. 2.0000 g) were used to measure the equilibrium moisture content (EMC) at the one of three constant temperatures (20, 25, and 30 °C) over an ERH range of 10–90%, using deionised water to produce the humidity and high-purity nitrogen to blow samples dry, preventing the growth of mold. The interval between gravimetric cycles was set at 10 min. The adsorption measurement cycle was started at 10% RH and first raised with a 10% RH step to 20% RH. Then, ERH was raised successively to 30%, 40%, 50%, 60%, 70%, 80%, and 90%. Desorption measurement cycle was performed from 80% to 10% RH using a 10% RH step and seven steps. The time per cycle had a minimal value of 50.0 min and a maximal value of 50.0 h. The default weight limit is +100%, and balance bandwidth (dm/dt) is ±0.01%/40 min. In the measurement cycle, the samples were self-actingly weighed on a balance (0.00001 g). The recorded data were treated by SPS-Toolbox Basic Rel. 1.15 software.

### 2.3. Fitting the Moisture Sorption Isotherms with EMC Equations

The experimental EMC data was produced as isotherm curves by Kaleidagraph version 4.541 software [17], with ERH (*a*_w_ × 100) and EMC data, respectively, entered onto the x- and y-axis. The EMC equations in Table 2 were used to fit the measured moisture isotherms of peony samples.

Fitting was performed by a non-linear regression analysis in SPSS v17.0 for Windows [23]. The criteria used for assessing the equation for the EMC/ERH data were the determination coefficient (*R*^2^), residue sum of squares (*RSS*), standard error (*SE*), mean relative percentage error (*MRE*), and residual plot. They were calculated using Equations (1)–(4), respectively.

(1) $$R^{2}=1-\sum_{i=1}^{n}{\left(m_{i}-m_{pi}\right)}^{2}/\sum_{i=1}^{n}{\left(m_{i}-m_{mi}\right)}^{2}$$

(2) $$RSS=\sum_{i=1}^{n}{\left(m_{i}-m_{pi}\right)}^{2}$$

(3) $$SE=\sqrt{\sum_{i=1}^{n}{\left(m_{i}-m_{pi}\right)}^{2}/\left(n-1\right)}$$

(4) $$MRE\%=\frac{100}{n}\sum_{i=1}^{n}\left|\frac{m_{i}-m_{pi}}{m_{i}}\right|$$

where *m*_i_ is the measured value, *m*_pi_ is the predicted value, *m*_mi_ is the average of test values, and *n* is the number of observations [24]. The fit of an equation to the EMC data of a sample was considered satisfactory if the *MRE* was lower than 10% [25]. If the plot of the measured EMC versus residual is random, the EMC equation is better. If the EMC versus residual plot is patterned, the EMC equation is worse [8].

Classification of the moisture isotherm types of the peony samples was performed based on the generalized D’Arcy and Watt (GDW) model provided by Furmaniak et al. [26]. In the GDW model (Table 2), a is the maximum sorption value (*M*_0_) on the primary centers and d is a parameter determining the ratio of molecules bonded to primary centers to those converted into secondary ones. b and c are kinetic constants. For type II (sigmoidal) and type III isotherms, parameter d > 0 and b is bigger or less than unity, respectively.

The hysteresis degree between adsorption and desorption curves at a certain temperature was calculated using Equation (5),

(5) $$Hysteresis\;degree\;\left(\%\right)=\frac{Desorptive\;EMC-Adsorptive\;EMC}{Adsorptive\;EMC}\times 100$$

### 2.4. Analysis of the Isosteric Heat of Sorption

The isosteric heat of moisture sorption (*h_s_*) is the amount of energy used to change one unit mass of a product from liquid to vapor at a certain temperature and ERH [8]. The *h_s_* for peony seed and cake samples was assayed using the below Equations [8]:

(6) $$\frac{h_{s}}{h_{v}}=1+\frac{P_{s}}{ERH}\cdot \frac{dt}{dP_{s}}\cdot {\left.\frac{\partial ERH}{\partial t}\right|}_{M}$$

(7) $$h_{v}=2501.33-2.363t$$

(8) $$P_{s}=\frac{6\times 10^{25}}{{\left(273.15+t\right)}^{5}}\cdot exp(-\frac{6800}{t+273.15})$$

(9) $$\frac{{dP}_{s}}{dt}=\frac{P_{s}}{\left(t+273.15\right)}\cdot (\frac{6800}{t+273.15}-5)$$

(10) $${\left.\frac{\partial ERH}{\partial t}\right|}_{M}=\frac{a\cdot ERH}{{\left(T+b\right)}^{2}}\cdot exp(-c\cdot M)$$

(11) $${\left.\frac{\partial ERH}{\partial t}\right|}_{M}=\frac{b\cdot ERH}{M^{c}}\cdot exp(a+b\cdot t)$$

where *h_v_* is the latent heat value of free water vaporization (kJ/kg), *h_s_* is the isosteric heat value of moisture sorption (kJ/kg), *M* is the equilibrium moisture content (% wet basis), *t* is the temperature, and $P_{s}$ is the saturated vapor pressure (Pa). Equation (6) can calculate the *h_s_*-to-*h_v_* ratio from ${dP}_{s}/dt$ and ${\left.\frac{\partial ERH}{\partial t}\right|}_{M}$, which was calculated using Equations (9) and (10), respectively. The *h_v_* in Equation (7) depends on the temperature. The *P_s_* was analyzed using Equation (8). The ${\left.\frac{\partial ERH}{\partial t}\right|}_{M}$ term is dependent on the sorption isotherm equation adopted; this study adopted the modified Chung-Pfost equation (MCPE, 10) and modified Halsey equation (MHAE, 11). a, b, and c are the parameters of MPCE and MHAE in the form of ERH=f(M,t).

### 2.5. Scanning Electron Microscopy (SEM)

Samples were set at the sample stage with double-sided conductive glue and then sputtered with gold under a gold particle sprayer (JEC-3000FC, Japan Electronics Co., Ltd., Tokyo, Japan). The samples were then set on the holder of a scanning electron microscope (JSM-IT 700HR, Japan Electronics Co., Ltd.) and observed using an accelerating voltage of 25 kV with 100 to 2000× magnification.

### 2.6. Data Analysis

SPSS software (Version 17.0, SPSS Inc. 2017 [23]) was used for data analysis. Independent-samples *t*-tests and one-way analysis of variance (ANOVA) were used to compare multiple means. Statistical significance was at *p* < 0.05.

## 3. Results

### 3.1. The Measured Moisture Sorption Isotherms of Peony Samples

In China, peony seeds with hulls, which are usually placed in nylon mesh bags, are stored in a ventilated, cool room for no longer than one year before pressing edible oils [1]. Thus, the moisture adsorption and desorption isotherms were determined in this study for ten unshelled peony seeds and one cake after oil pressing. It took about 29 days to determine the moisture adsorption/desorption isotherms of each batch of samples by the dynamic moisture sorption apparatus. Because of the long measuring time, we measured the isotherm at 20, 25, and 30 °C, which are very useful for the drying, storage, and packaging of peony seeds and cake.

In the dynamic moisture sorption apparatus, the RH inside the climatic chamber is conditioned via mixing a dry nitrogen gas flow and the gas flow saturated with water. The instrument has a dew point analyzer and a microbalance for the accurate determination of RH and weight, respectively [27]. Water sorption is obtained by measuring the variation in mass of a sample that is caused by the change in ambient RH. We used the equilibrium criterion (dm/dt limit 0.01%/40 min) and measured the 10–90% ERH isotherm because the sorption curves show large variation in the relative mass in the quasi-steady-state regime for the RH steps above 80% [27].

**Figure 3:**
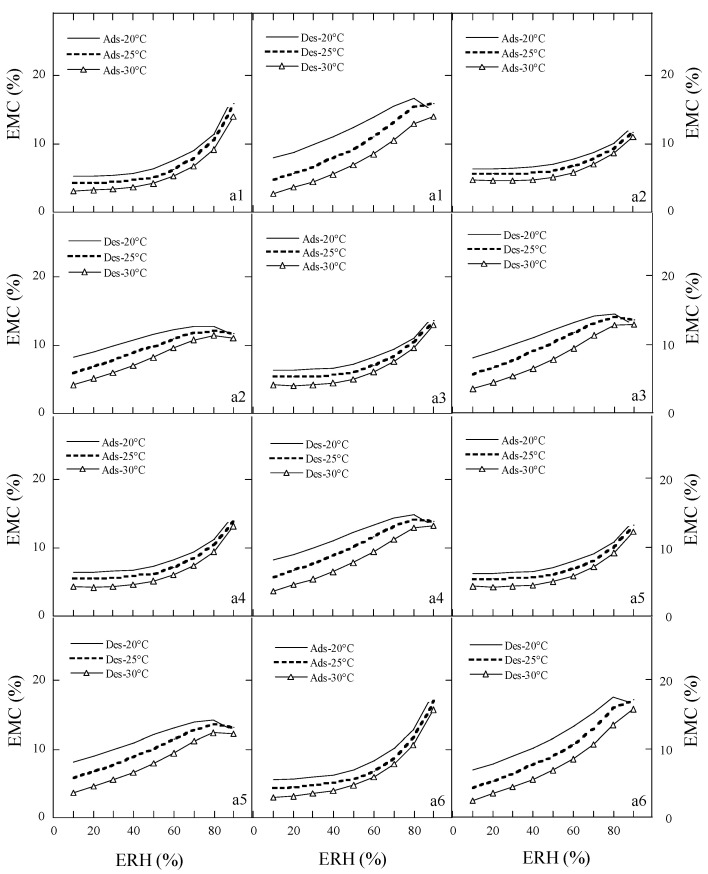
Adsorption and desorption isotherms of samples (**a1**–**a6**). Note: Number of repetitions―*n* = 2 for each sample.

**Figure 4:**
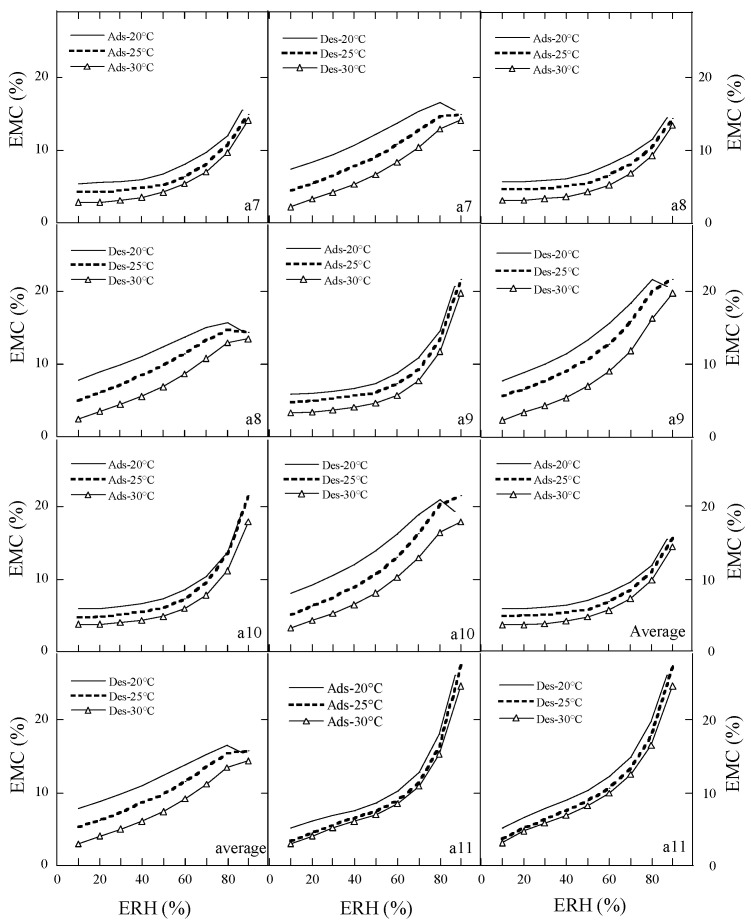
Adsorption and desorption isotherms of samples (**a7**–**a11**) and average data. Notes: Number of repetitions―*n* = 2 for each sample; the average is the mean of the EMC/ERH data of ten species of peony seeds.

According to the Brunauer’s classification on the moisture sorption isotherms [8], the moisture adsorption and desorption isotherms of the ten species of peony seeds were type II (sigmoidal) or type III-like (Figure 3 and Figure 4). The adsorption and desorption isotherm of sample a11 (Ziyun Mudan cake) showed type III-like and type II curves, respectively. Temperature and ERH had a strong impact on the adsorption and desorption curves. As the temperature increased, the EMC decreased at constant ERH, while at constant temperature, the EMC increased as ERH increased. The equilibrium moisture contents (EMCs) of peony samples at constant ERH decreased as the sorption temperature increased, because the kinetic energy of water molecules is higher and water adsorption is lower at higher temperatures. With the increase in the mobility of the water molecules, the water molecules could not bind to the samples through hydrogen bonds, thereby the moisture content of the samples decreased with an increase in temperature [28].

There was hysteresis between the desorption and adsorption isotherms of the peony samples. At 90% ERH and 25 °C, the desorption EMCs of the ten species of peony seeds were 11.62–21.65% with an average of 15.72%, but the EMC of peony cake (sample a11) was 27.47%, suggesting that the residual cake after oil pressing had a very high water adsorption capacity due to its hydrophilic proteins and polysaccharides and absence of a seed coat.

### 3.2. The Optimal Equation of Moisture Sorption Isotherms of Peony Samples

The EMC equations in Table 2 were adopted to fit the adsorption/desorption isotherms of the 11 peony seed samples in the range of 20 to 30 °C. Their average statistical parameters are provided in Table 3. When the determination coefficient (*R*^2^) was larger and other parameters like *RSS*, *SE*, and MRE were smaller, the EMC equations were a better fit for the EMC data. In the form of $M=f\left(ERH,t\right),$ for adsorption, the equations with the highest to lowest fit were Poly, MHAE, MOE, Peleg, 4-Para, GDW, MGAB, MCPE, and MHE. For desorption, the ranking of the equations from highest to lowest fit was Poly, MCPE, MHE, MOE, MHAE, Peleg, 4-Para, GDW, and MGAB. In the form of ERH=f(M,t), the equations with the highest to lowest fit were MHAE, MOE, MCPE, MHE, and MGAB for adsorption, and MCPE, MOE, MHAE, MHE, and MGAB for desorption. Thus, Poly, MHAE, and MOE were selected as the optimal equations to fit the EMC data of the peony seed samples. The seven-parameter polynomial can be used for moisture prediction in bulk peony seeds, while the three-parameter reversible equations like MHAE and MOE are used for calculating isosteric heats of sorption.

Table 4 shows the average statistical parameters of the EMC equations fitting the adsorption/desorption isotherms of a peony cake sample in the range of 20 to 30 °C. In the form of $M=f\left(ERH,t\right),$ for adsorption, the equations with the highest to lowest fit were MHAE, Poly, MGAB, Peleg, 4-Para, GDW, MOE, MHE and MCPE. For desorption, the equations from highest to lowest fit were MHAE, Poly, MOE, Peleg, 4-Para, GDW, MGAB, MHE, and MCPE. In the form of ERH=f(M,t), the equations with the highest to lowest fit were MHAE, MOE, MGAB, MCPE, and MHE for adsorption, and MOE, MHAE, MGAB, MCPE, and MHE for desorption. Therefore, Poly, MHAE, and MOE were also selected as the optimal equations to fit the EMC data of the peony cake sample.

Table 5 and Table 6 show the polynomial parameters and key statistical parameters. The *R*^2^ and *MRE* values ranged from 0.9888 to 0.9986 and 0.83 to 6.52% for adsorption, and 0.9816 to 0.9964 and 1.36 to 4.81% for desorption, respectively. The EMC residual plots fitted by polynomial were all random distributions for the desorption and adsorption data of ten varieties of peony seed and one peony cake (Figure S1).

Table 7 shows the MHAE parameters and key statistical parameters. The *R*^2^ and *MRE* values ranged from 0.9417 to 0.9949 and 4.48 to 11.53% for adsorption, and 0.7815 to 0.9973 and 4.18 to 17.93% for desorption, respectively. The EMC residual plots fitted by MHAE were random distributions for the adsorption data of ten varieties of peony seed and the adsortion/desorption data of one peony cake, but were patterned distributions for the desorption data of ten varieties of peony seed (Figure S2).

MHAE is usually used to fit the EMC data of oil seeds, but its key statistical parameters for the EMC/ERH data of the peony seeds were inferior to those of polynomial (Poly), although it was a good fit for the EMC data of the peony cake. Thus, Poly is recommended as the most suitable EMC equation for peony seeds and cake.

### 3.3. The Classification Moisture Sorption Isotherms of Peony Samples

The parameters in the GDW equation were used for the moisture isotherm types. For type II (sigmoidal) and type III isotherms, parameter d > 0 and b is larger or less than unity, respectively. The samples such as a1, a2, a4, and a8 had type II adsorption curves, but the other seven samples (a3, a5, a6, a7, a9, a10, and a11) had type III-like adsorption curves (Table 8). Among the 11 samples, three samples (a1, a10, and a11) had type II desorption curves, four samples (a6, a7, a8, and a9) had type III-like desorption curves, and the other four samples (a2, a3, a4, and a5) were judged by us to show the type II-like desorption curves. The classic static gravimetric method could easily obtain the whole sigmoidal curve of moisture sorption in cereals and oilseed, but the dynamic determination method for the moisture sorption of biomaterials could easily obtain the linear portion of a sigmoidal curve [29,30]. Although many adsorption or desorption isotherms of peony samples in the present study were classified into the type III-like curves, they essentially should be type II (sigmoidal) curves because the sample was sensitive to changes in RH when the RHs in the climatic chamber were above 80%, in combination with the fact that the dynamic sorption analyzer was not able to control the RH in this RH regime well enough [27].

### 3.4. The Moisture Sorption Hysteresis of Peony Samples

**Figure 5:**
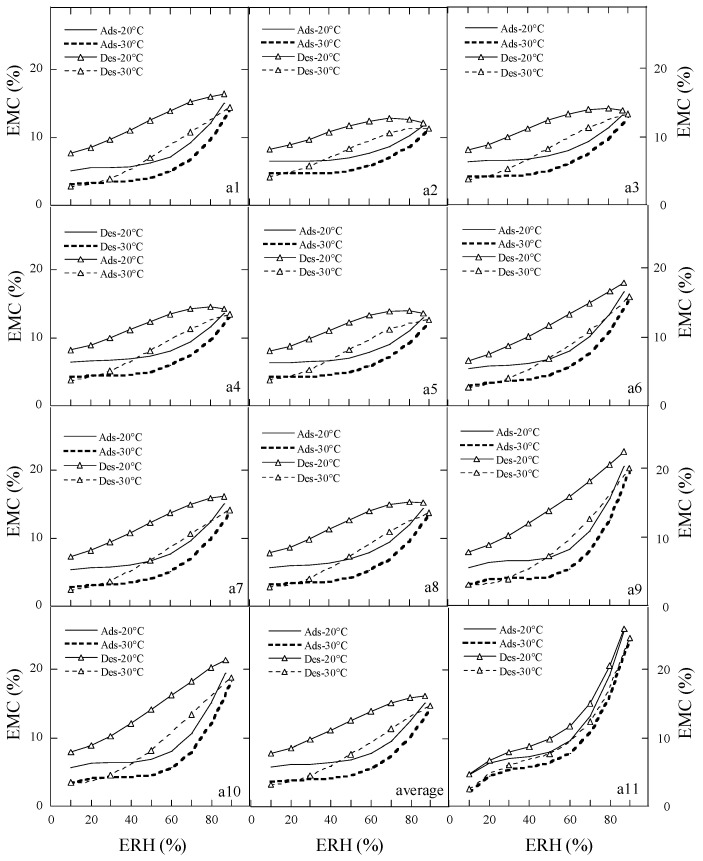
The predicted moisture sorption isotherms of peony samples using polynomial. Note: (**a1**–**a11**) are the samples; the average is the mean of the EMC/ERH data of ten species of peony seeds.

Sorption hysteresis is a fundamental physical property of the food material [8]. The predicted moisture sorption isotherms of the peony samples using Poly are shown in Figure 5. There was a relatively evident hysteresis loop between the desorption and adsorption isotherms in the a2, a3, a4, a5, a9, and a11 samples, and the hysteresis loop became smaller as the temperature increased. The hysteresis behavior can be explained by capillary condensation and swelling fatigue theory; capillary condensation, pore closure, and changes in cellular structure elasticity might contribute to hysteresis [8]. The decrease in hysteresis loop with increasing temperature can be demonstrated by monolayer and multi-layer sorption theory [8].

**Figure 6:**
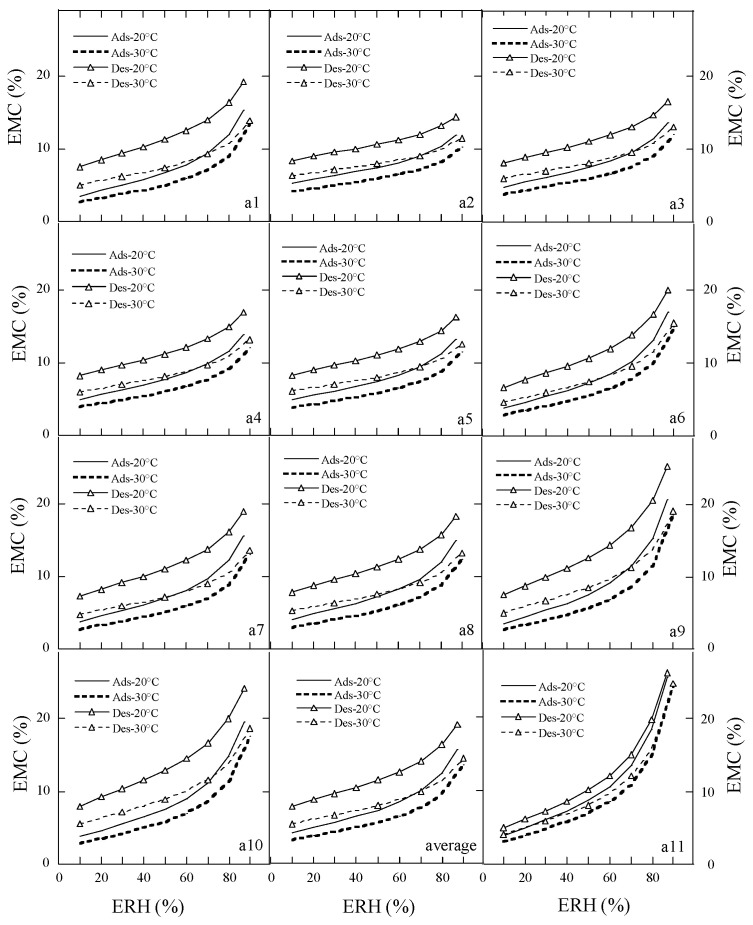
The predicted moisture sorption isotherms of peony samples using MHAE. Note: (**a1**–**a11**) are the samples; the average is the mean of the EMC/ERH data of ten species of peony seeds.

The predicted moisture sorption isotherms of the peony samples using MHAE are shown in Figure 6. There was a pronounced hysteresis phenomenon between the desorption and adsorption isotherms for the peony seeds (a1–a10 samples), while there was a clear hysteresis loop for the cake (a11 sample). The hysteresis phenomenon decreased as the temperature increased. This trend might be attributed to an increase in the elasticity of the capillary walls and the higher capacity to form hydrogen bonds between the dry matter and moisture present in peony seeds or cake [31].

**Figure 7:**
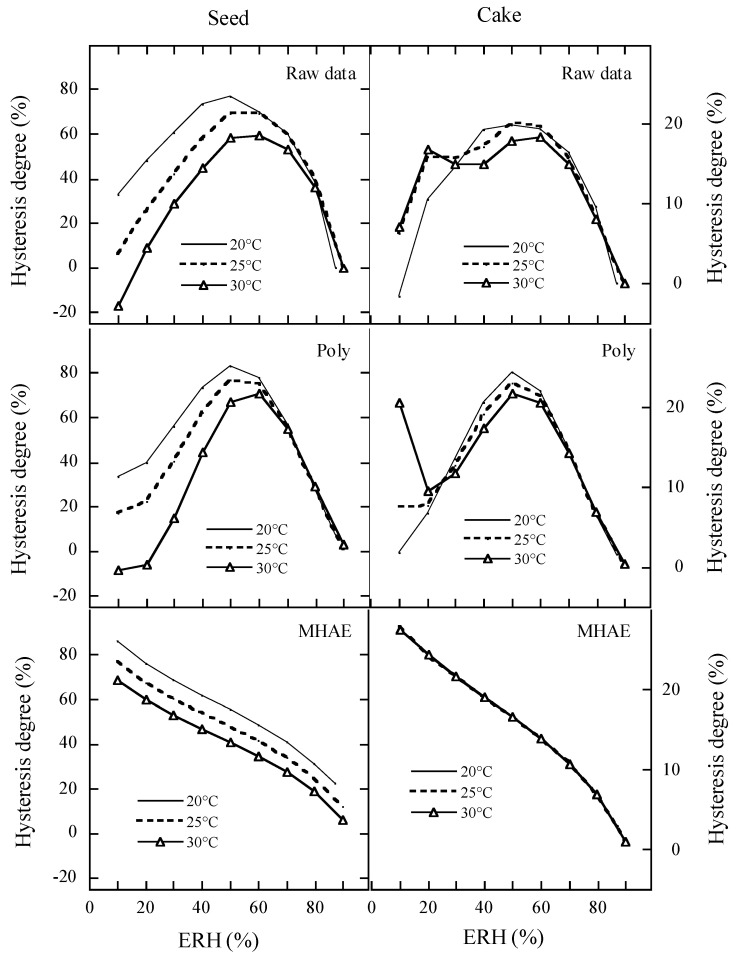
Comparison of the hysteresis degree of moisture adsorption in peony seeds and cake among raw data, Poly, and MHAE.

Figure 7 further shows the hysteresis degree of moisture adsorption in peony seeds and cake. The raw data and Poly-predicted data almost show parabola with inflection point at 50% ERH for the hysteresis degree of moisture adsorption in peony seeds and cake, respectively, while the MHAE-predicted hysteresis degree almost linearly decreased with increasing ERH at certain temperature. The hysteresis degree curves predicted by MHAE decreased with increasing temperature for peony seeds, but cannot distinguish the effect of temperature for peony cake. Thus, we conclude that the Poly-predicted hysteresis degree could represent the origin hysteresis degree of peony seeds and cake. Considering that Poly contains the temperature-ERH interaction like ${ERH}^{2}t+ERH\cdot t$, and clearly outperformed other EMC models numerically, we recommend Poly for the EMC/ERD data of peony seeds and cake. However, the closed structure of this seven-parameter model to physical interpretation and the potential risk of overfitting will be further studied.

### 3.5. The Monolayer Moisture Content of Peony Samples

The monolayer moisture content (*M*_0_) is considered as an ideal moisture content to avoid changing the product’s quality, such as lipid oxidation [32], which was estimated by the parameter a in MGAB (Table 9) and GDW (Table 8) models in the present study. The adsorption *M*_0_ in peony seed samples estimated by MGAB was in the range of 3.12–4.09% w.b., being averaged as 3.64 ± 0.42%, while the desorption *M*_0_ was in the range of 5.39–6.82% w.b. with an average value of 6.21 ± 0.47%. The adsorption and desorption *M*_0_ in the peony cake were 4.28% and 4.83%, respectively. These results suggested that the desorption *M*_0_ in peony samples is higher than the adsorption *M*_0_. The adsorption *M*_0_ values in peony samples were all below the maximum monolayer moisture content (9.1% w.b.) given by Labuza [33] and Gül et al. [32]. The adsorption *M*_0_ values in peony samples were comparable to those of flax seed (4.13% w.b.), sunflower seed (2.91%), and safflower seed (4.28%), which were analyzed by MGAB to the EMC/ERH data in range of 11–96% ERH and 10–55 °C [11]. The adsorption–desorption *M*_0_ difference in peony samples might arise from the microstructure, such as changes in water binding sites, and lipid–protein interaction.

In case of the *M*_0_ estimated by GDW (Table 8), the adsorption *M*_0_ in peony seed samples was in the range of 3.44–5.05% w.b. and was averaged as 4.14 ± 0.60%, while the desorption *M*_0_ was in the range of 3.28–4.29% w.b. with an average value of 3.86 ± 0.38%. The adsorption and desorption *M*_0_ in the peony cake were 3.68% and 4.00%, respectively. The average *M*_0_ of adsorption estimated by GDW in peony seed samples was similar to that of desorption, maybe due to the hypothesis that the m_0_ in the GDW equation is the maximum sorption value on primary centers (equal to their concentration) [26].

### 3.6. The Safe Storage MC of Peony Samples

The desorption polynomial equation was used to predict the absolutely safe storage MC of the peony samples (Table 10), which indicates no mold growth. At ERH = 65% and 25 °C, the predicted MC of the peony seeds ranged from 11.38 to 14.66%, while at ERH = 60% and 25 °C, the predicted MC ranged from 10.99 to 13.47%. Both of these results were higher than the 5.54–6.82% MC range of the initial samples. The mean safe MCs for ten species of peony seeds at 25 °C were predicted to be 11.69% and 12.48% for ERH = 60% and ERH = 65%, respectively. The absolutely safe MC for peony cake (a11) at 25 °C was predicted to be 10.54% and 11.92% for ERH = 60% and ERH = 65%, respectively. At 25 °C and ERH = 65%, the absolutely safe MC of peony seeds (12.48%) is higher than that of soybeans (10.16%) [34], but peony cake (11.92%) and soybean cake (11.64%) [35] have similar absolutely safe MCs.

### 3.7. The Isosteric Heat of Sorption of Peony Samples

**Figure 8:**
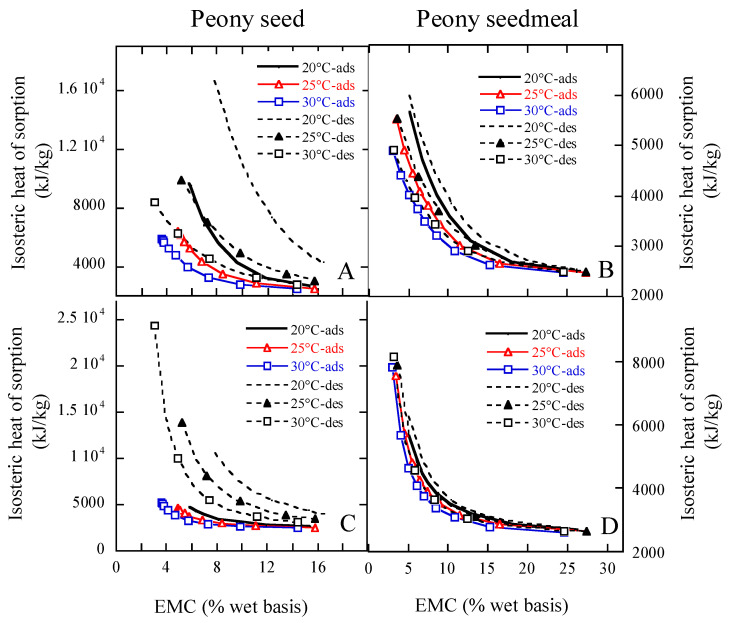
Comparison of adsorption (ads) and desorption (des) isosteric heats of peony samples at different temperatures predicted by the MCPE and MHAE. Notes: (**A**,**C**), MCPE; (**B**,**D**), MHAE.

The coefficients a, b, and c of the MCPE and MHAE in a form of ERH=f(M,t) (Table 11) were adopted to analyze the sorption isosteric heats. Figure 8 shows the influence of moisture content (MC) on desorptive and adsorptive isosteric heats of peony samples. In the case of MCPE, the isosteric heats for both desorption and adsorption of peony seed (Figure 8A) decreased quickly with the increase in the sample MC until a MC of 12% and 11% w.b. were, respectively, achieved, but above these values, they decreased slowly with increasing moisture content. At lower MCs below these values, the isosteric heats of both desorption and adsorption of peony seeds at lower temperatures were greater than those at higher temperatures. The isosteric heats of peony seed desorption were greater than those of adsorption below 13.5% MC, but thereafter, there was minimal difference found between desorption and adsorption (Figure 8A). The isosteric heats for both desorption and adsorption of peony cake (Figure 8B) decreased quickly with the increase in the sample MC until a MC of 17.5% w.b. was reached, but above 17.5%, they decreased smoothly with the increase in MC.

When MHAE was used to analyze the isosteric heats of sorption, the isosteric heats for both desorption and adsorption of peony seeds (Figure 8C) decreased rapidly with the increase in the sample MC until the moisture content of 11% and 10% w.b. was, respectively, reached, but above these values, they decreased steadily with the increase in MC. The isosteric heats for both desorption and adsorption of peony cake (Figure 8D) decreased rapidly with the increase in the sample MC until a moisture content of 17.5% w.b. was achieved, but above 15%, they decreased steadily with the increase in MC. These results show that the heat of sorption of peony seeds and cake approached that of pure water at about 11% and 15% w.b. MC, respectively, because MHAE was better to fit the EMC/ERH data of peony seeds and cake than MCPE.

The rapid rise in the isosteric heat of sorption at low MC might be due to existing substantially active polar sites on the surface of the peony seed and cake samples, which were covered by water molecules producing a mono-molecular layer [8]. The decrease in the isosteric heats with larger amounts of sorbed water can be quantitatively explained by considering that sorption develops initially on the most active available sites realizing high interaction energy. Once these sites were occupied, sorption happens on the less active ones, forming lower heats of sorption [8]. In low MCs, the amounts of the isosteric heats were greater than the latent heat of water vaporization, indicating that the binding energy of the water molecules and the sorption sites was greater than the energy which maintains the pure water molecules together in the liquid stage [36]. At high MCs, no appreciable difference was found between the sorption isosteric heat and the latent heat of water vaporization in the broad range of MCs. In the present study, the isosteric heat of sorption of peony seeds and cake approached that of pure water around 11% and 15% w.b. MC, respectively. These isosteric heats can be adopted in modeling energy consumption and drying calculation in the drying process of peony seed and cake products, ensuring the safe storage of these products while maintaining their original quality to the greatest extent possible.

### 3.8. The Outer Shell of the Peony Seed and the Microstructure of Peony Kernels

Peony seeds are solid and tough, with the outer shell having a glossy black-brown surface and an off-white inner surface (Figure 1). The seed surfaces have slight protuberances, depressions, patterns, or a waxy layer on the surface. The seed coat consists of 4–5 layers of cells. The first is the epidermis, a compressed thin layer of thick-walled cells. The second is a layer of shriveled thin-walled cells. The third is a layer of thick-walled, partially lignified cells. The fourth is a narrow layer of colored matter, beneath which are the cells of the aleurone layer, which are generally included in the seed coat [37,38,39].

**Figure 9:**
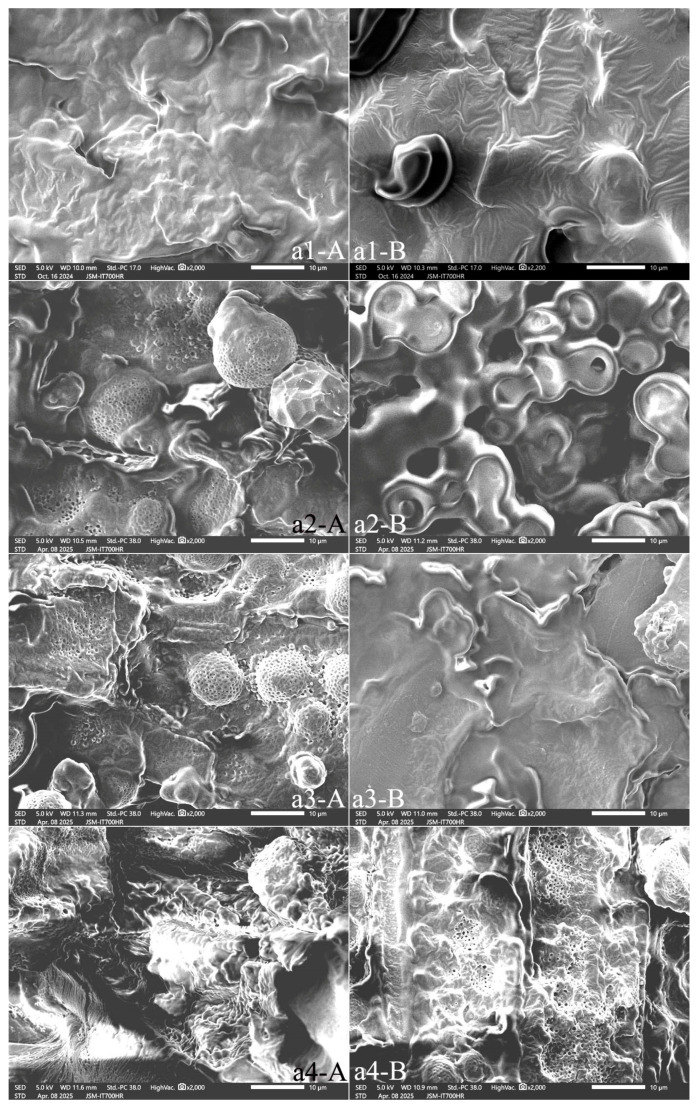
SEM of the lengthwise section (**A**) and cross section (**B**) of the samples (**a1**–**a4**). Note: All the photos were enlarged at 2000×.

**Figure 10:**
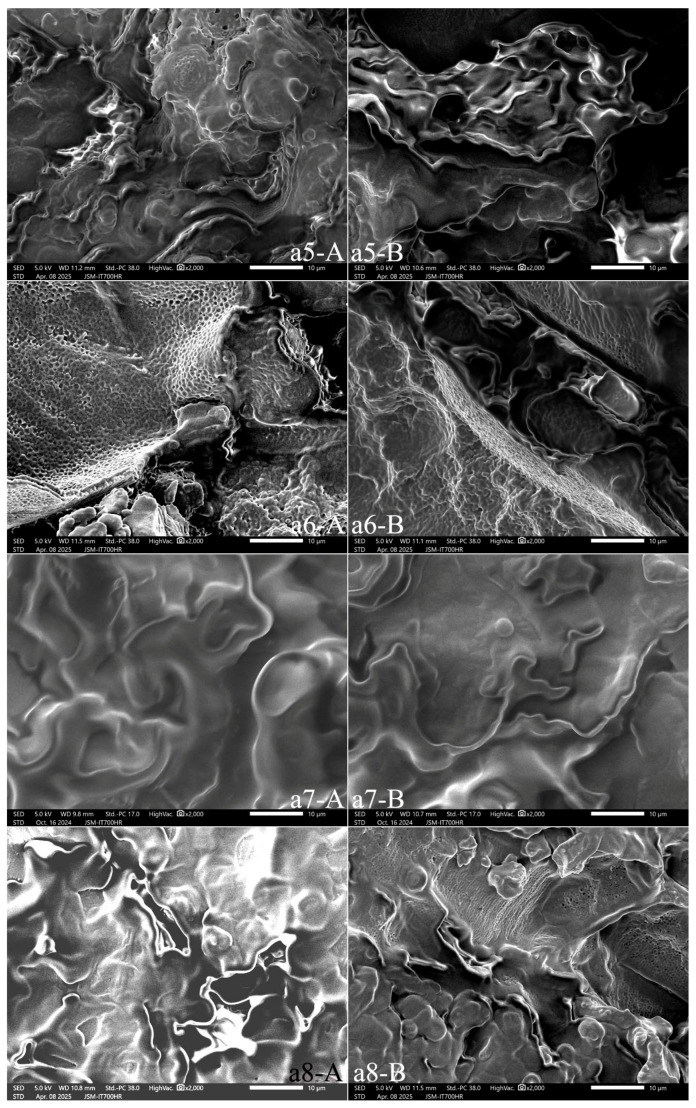
SEM of the lengthwise section (**A**) and cross section (**B**) of the samples (**a5**–**a8**). Note: All the photos were enlarged at 2000×.

**Figure 11:**
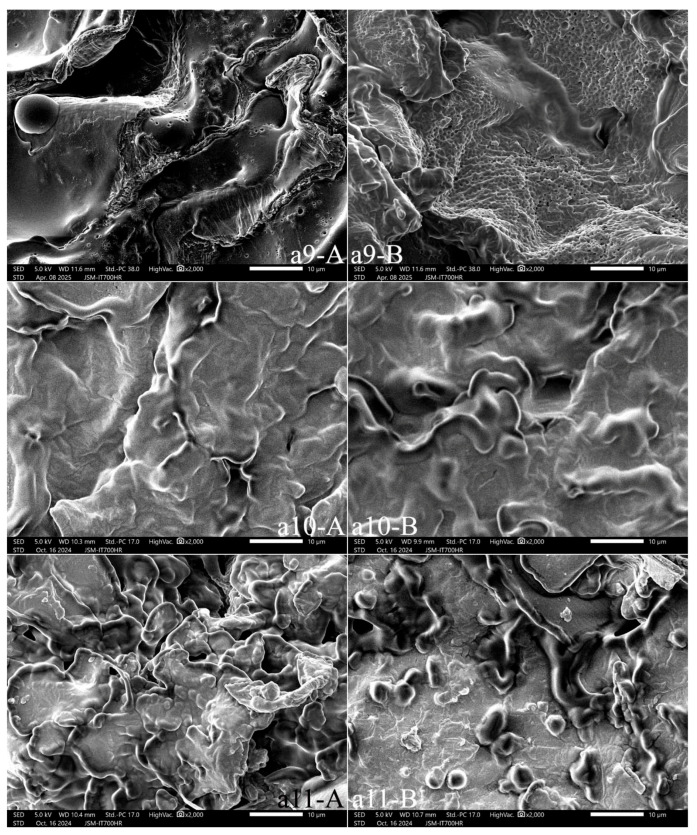
SEM of the lengthwise section (**A**) and cross section (**B**) of the samples (**a9**–**a11**). Note: All the photos were enlarged at 2000×.

A peony seed comprises high contents of lipid (31%) and protein (20.4%) [4]. It follows then that the oil bodies and protein bodies are the main components in the cells of peony seed. The lengthwise sections of samples a2, a3, a4, a6 and a9 revealed spherical, porous lipid bodies with cilia (Figure 9(a2-A,a3-A,a4-A); Figure 10(a6-A); and Figure 11(a9-A)). Samples a1, a5, a7, a8, and a10 had smooth-surfaced protein bodies and oil bodies (Figure 9(a1-A), Figure 10(a5-A,a7-A,a8-A) and Figure 11(a10-A)). The cross sections of samples a4 (Figure 9(a4-B)), a6 (Figure 10(a6-B)), and a9 (Figure 11(a9-B)) revealed that the porous bodies with cilia were spherical lipid bodies, while the other samples (Figure 9(a1-B,a2-B,a3-B), Figure 10(a5-B,a7-B,a8-B) and Figure 11(a9-B,a10-B)) contained smooth-surfaced protein bodies and oil bodies.

The SEM images of the lengthwise and cross sections of peony kernel samples showed that the cell wall enclosed substances such as oils, proteins, etc., within the cell. The oils and their associated particles existed as tiny-diameter spherical liposomes, and the oil within the cell was in the form of thousands of spherical liposomes, which were attached to the inner surface of the cell and to the outer surface of the protein-storing vacuoles. SEM images of their lengthwise and cross sections showed that samples a1, a5, a7, a8, and a10 had accumulated lamellar gel structures comprising stored protein, lipid, starch, and cell wall polysaccharides.

Figure 11(a11-A,a11-B) show that the peony cake was rich in protein bodies but contained fewer oil bodies. After cold pressing the oil in peony seeds, the dry cake formed a block containing thousands of spherical protein bodies in vacuoles, as well as cell debris.

The absolutely safe storage MC (13.47%) of yellow peony seed (a10) at 25 °C was higher than that of a2 (10.99%) and Ziyan Feishuang peony seed (a7, 11.12%). This may have been due to a higher amount of hydrophilic materials like protein and cell wall polysaccharides in the seed coat (possible thinner shell thickness) and endosperm cells, and higher protein/oil bodies ratios of the a10 sample. At ERH = 60% and 25 °C, the predicted absolutely safe storage MC of a11 (peony cake) was 10.54%, which was similar to soybean cake [35].

Oil-type peony cultivation in China is concentrated in the central plain regions, where the peony seeds mature in late summer and early autumn. When the pods are harvested, they are in an environment with high temperature and humidity, and their respiration is very vigorous. Under large-scale production conditions, management can easily lead to mold. Even if the moisture content is properly controlled, the incidence of browning in the seeds is still high during long-term storage, which can directly affect the quality of the peony seed oil [1]. The present study indicated absolutely safe moisture content at 60–65% ERH and 20–30 °C. At 25 °C and 65% ERH, the peony seed and cake MC values are 12.48% and 11.92%, respectively; these were close to the 12% threshold for peony seeds and cake in China’s national standards, LST 3310–2017 [40] and LST 3120–2019 [41].

The moisture sorption isotherms of important global oil crops have been reported for peanuts [42,43], soybeans [8,44], sesame [44,45], sunflower seeds [46] and rapeseed [47,48]. The present study provides the first report of EMC/ERH data for peony seeds. The American Society for Agricultural and Biological Engineers (ASABE) recommends the MHAE to fit the moisture sorption isotherms of oilseeds like rapeseed, cotton seeds, peanut kernels, soybeans, safflower seeds, and sunflower seeds [11]. In the present study, the *R*^2^ and *MRE* values of MHAE for peony seeds and cake were 0.9417–0.9949 and 4.48–11.53% for adsorption, and 0.7815–0.9973 and 4.18–17.93% for desorption, respectively. However, the *R*^2^ and *MRE* values of Poly for peony seeds and cake were 0.9888–0.9986 and 0.83–6.52% for adsorption, and 0.9816–0.9964 and 1.36–4.81% for desorption, respectively. Moreover, the Poly-predicted hysteresis degree curves were almost similar to the raw hysteresis degree curves. Therefore, the developed polynomial provided the best fit for the EMC data of peony seeds and cake, which can be used for the smart detection of moisture content in bulk peony seeds or cake.

The four equations like MCPE, MHE, MOE, and MHAE recommended by ASABE [11] show the logarithmic or exponential relationship between intergranular ERH and temperature in the food materials, but do not show the interaction between the quadratic term and linear term of ERH and the temperature. The prospects have been bright for the online detection of moisture content in bulk cereals or seeds by using the common equilibrium moisture equation and the intergranular sensors of temperature and relative humidity, but the road is very challenging [12,49]. Our developed seven-parameter polynomial in the present study shows the effect of the intergranular ERH and the ambient air temperature, especially the interaction effect between the quadratic term and linear term of ERH and temperature, and is more likely to achieve the online moisture detection and storage optimization in bulk food materials using the intergranular sensors of temperature and relative humidity, implementing an equilibrium moisture cable to replace the existing temperature cable in bulk cereals and seeds.

Jirayucharoensak et al. [50] calculated the suitable water activity conditions for the storage of inulin samples using the Lewicki-3 model and found that the moisture content of inulin powder should be kept at ≤5.75% wet basis (w.b.) during storage, and the ambient relative humidity at 20 and 30 °C should not exceed 18.32% and 37.12%, respectively. When the monolayer moisture content (*M*_0_) of peanut pod was 4.55% w.b., its adsorption ERHs at 20, 25, and 30 °C calculated using the MHAE were 14.45%, 16.46%, and 18.60%, respectively, while the adsorption ERHs of peanut kernel at 20, 25, and 30 °C calculated using *M*_0_ = 3.35% and the MHAE were 11.00%, 11.53%, and 12.06%, respectively [43]. In the present study, when the *M*_0_ of peony seeds was 3.55% w.b., its adsorption ERHs at 20, 25, and 30 °C calculated using the MHAE were 15.09%, 33.39%, and 52.92%, respectively, which is in contrast to the results of inulin samples [50] and peanut pods [43]. The adsorption ERHs of the peony cake samples at 20, 25, and 30 °C were 9.57%, 15.5%, and 22.73%, respectively, using *M*_0_ = 4.28% w.b. and the MHAE. These results suggest that peony seeds and cake should be kept in packaging that can prevent moisture transfer from the surrounding air into the package, especially in subtropical regions where the relative humidity of the environmental air is usually high. If the moisture content in raw peony seed or cake is relatively high, natural or mechanical aeration should be carried out.

Transmission electron microscopy of the microstructure of rapeseed and cashew kernels revealed that the pores (plasmodesmata) in their cell walls were channels through which oil flowed out of the seed cell during cold extrusion [37]. Zhao and Wu [39] first observed the accumulation of oil bodies in the kernels of tree peony seeds using transmission electron microscopy. The present study used SEM to observe lengthwise and cross sections of peony kernels surfaces. Compared with the peony kernel cake, which was rich in protein bodies, the peony kernels of a1, a2, a5, a8, and a10 had easily observable protein bodies that contributed to their higher moisture content, especially the seeds of Huang Mudan peony (a10). The present study measured the moisture adsorption/desorption isotherms of peony seeds because they are stored in hull form until being pressed into oil. The hard hulls have an important effect on moisture sorption. Further investigation of the moisture sorption isotherms of peony seed hulls and kernels, and the analysis of biochemical component contents, will elucidate the sorption hysteresis of peony kernels.

## 4. Conclusions

A tree of oilseed peony can produce oilseed for 30 to 60 years at six tonnes per hectare, which would help solve the problem of insufficient edible oil for human consumption in 2050. This study determined the two key factors impacting its safe storage: moisture content and temperature. Both peony seeds and cake had a type II (sigmoidal) or type III adsorption and desorption isotherm. The optimal equations fitting the isotherms of peony samples were our developed seven-parameter polynomial (Poly), MHAE, and MOE. Poly was superior to MHAE in fitting the peony sample moisture sorption isotherms. The absolutely safe storage moisture level predicted by Poly at 25 °C and 65% ERH was 12.48% for seeds and 11.92% for cake. The desorption monolayer moisture content (M_0_) in peony seeds and cake estimated by MGAB were 6.21 ± 0.47% and 4.83%. The isosteric heat of sorption of peony seeds and cake approached that of pure water at about 11% and 15% w.b. MC, respectively. Microstructural analysis showed that the peony seeds were rich in liposomes, which were attached to the inner surface of the cell wall and the outer surface of the protein storage vacuole. The cake was rich in protein bodies and hydrophilic polysaccharides. This study is beneficial for the safe storage and aeration management of bulk peony seeds. Poly is likely to use the online detection of moisture content in bulk food materials using temperature and relative humidity sensors.

## Figures and Tables

**Table 1:** The moisture content (MC) of peony samples.

Sample No	Species	Samples	Shape	Initial Moisture Content (% Wet Basis)
a1	2# peony	Seed	Oval	5.54 ± 0.03
a2	21# peony	Seed	Long oval	6.67 ± 0.01
a3	30# peony	Seed	Short oval	6.64 ± 0.14
a4	91# peony	Seed	Long oval	6.82 ± 0.14
a5	92# peony	Seed	Oval	6.58 ± 0.03
a6	Zishen	Seed	Subglobose	5.99 ± 0.01
a7	Ziyan Feishuang	Seed	Short ovoid;	5.72 ± 0.08
a8	Zhouban Bai	Seed	Circular	5.96 ± 0.06
a9	Dian Mudan	Seed	Flattened	6.35 ± 0.07
a10	Huang Mudan	Seed	Irregular	6.42 ± 0.01
a11	Ziyun Mudan	Cake	Block	7.15 ± 0.13

Notes: #, No.; Initial moisture content is expressed as mean ± SD, number of repetitions―*n* = 3.

**Table 2:** EMC equations used in this study.

Equation Name	Formula	Reference
GDW	$M=\frac{a\cdot b\cdot ERH}{1+b\cdot ERH}\cdot \frac{1-c\cdot \left(1-d\right)\cdot ERH}{1+c\cdot ERH}$	Furmaniak et al. [18]
MCPE	$ERH=exp\left[\frac{-A\cdot exp(-C\cdot M)}{B+t}\right]$ $M=\frac{1}{-c}ln\left[\frac{\left(t+b\right)\cdot ln\left(ERH\right)}{-a}\right]$	Li [8]
MGAB	$M=\frac{a\cdot b\cdot (c/t)\cdot ERH}{\left(1-b\cdot ERH\right)\cdot \left(1-b\cdot ERH+c/t\cdot b\cdot ERH\right)}$ $ERH=\frac{2+\frac{c}{t}\cdot \left(\frac{a}{M}-1\right)-{\left[{\left(2+\frac{c}{t}\cdot \left(\frac{a}{M}-1\right)\right)}^{2}-4\left(1-\frac{c}{t}\right)\right]}^{0.5}}{2B(1-\frac{c}{t})}$	Yanniotis and Blahovec [19]
MHAE	$M={\left[\frac{exp(a+bt)}{-lnERH}\right]}^{1/c}$ $ERH=exp\left[-\frac{exp\left(a+bt\right)}{M^{c}}\right]$	Luo et al. [20]
MHE	$M={\left[\frac{ln(1-ERH)}{-a\cdot \left(t+b\right)}\right]}^{1/c}$ $ERH=1-exp\left[-a\cdot (t+b)\cdot M^{c}\right]$	Li [8]
MOE	$M=\frac{a+bt}{{\left(1/ERH-1\right)}^{1/c}}$ $ERH=\frac{1}{1+{\left(\frac{a+bt}{M}\right)}^{c}}$	Luo et al. [20]
4-Para	$M=a+b\cdot ERH+c\cdot {ERH}^{2}+d\cdot {ERH}^{3}$	Li and Schmidt [21]
Peleg	$M=a\cdot {ERH}^{c}+b\cdot {ERH}^{d}$	Peleg [22]
Polynomial (Poly)	$M=a\cdot {ERH}^{3}+b\cdot {ERH}^{2}+c\cdot ERH+d\cdot {ERH}^{2}\cdot t+e\cdot ERH\cdot t+f\cdot t+g$	Li [8]

Notes: GDW, generalized D’Arcy and Watt; MCPE, modified Chung-Pfost equation; MGAB, modified Guggenheim–Anderson–de Boer; MHAE, modified Halsey; MHE, modified Henderson equation; MOE, modified Oswin; *M*, equilibrium moisture content (%); *ERH*, equilibrium relative humidity (decimal); and *t*, temperature (°C). The coefficient a in GDW and MGAB is monolayer moisture content (% w.b.); a, b, c, d, e, f, and g are the equation parameters.

**Table 3:** Comparison of the statistical parameters of the equations for the average EMC/ERH data of ten species of peony seeds.

Type	Sorption	Eqn.	RSS	SE	*R* ^2^	*MRE* (%)	Order
M=f(ERH,t)	ads	GDW	27.4911	1.1953	0.9099	13.9192	6
		MCPE	43.5814	1.8159	0.8829	15.9453	8
		MGAB	30.2073	1.2586	0.8970	14.4982	7
		MHAE	11.6489	0.4854	0.9617	8.7307	2
		MHE	49.4681	2.0612	0.8450	17.6949	9
		MOE	24.4611	1.0192	0.9211	12.5904	3
		4-para	25.5045	1.1089	0.9170	13.4375	5
		Peleg	25.0197	1.0878	0.9178	13.1018	4
		Poly	1.7878	0.0894	0.9962	2.4219	1
	des	GDW	94.8297	4.1230	0.7695	19.8856	8
		MCPE	43.2128	1.8004	0.8892	8.0295	2
		MGAB	116.2842	4.8452	0.7150	23.8874	9
		MHAE	56.4090	2.3508	0.8554	15.0050	5
		MHE	38.7306	1.6138	0.9045	12.0697	3
		MOE	43.2128	1.8004	0.8892	13.1843	4
		4-para	91.7127	3.9875	0.7788	19.8856	7
		Peleg	94.1037	4.0914	0.7707	19.8411	6
		poly	4.5823	0.2291	0.9915	3.2217	1
ERH=f(M,t)	ads	MCPE	0.1999	0.0083	0.8875	30.4239	3
		MGAB	0.3527	0.0147	0.8013	39.9156	5
		MHAE	0.1416	0.0059	0.9203	25.6129	1
		MHE	0.2489	0.0104	0.8599	34.3679	4
		MOE	0.1769	0.0073	0.9005	28.8790	2
	des	MCPE	0.0823	0.0034	0.9537	11.4745	1
		MGAB	0.1718	0.0072	0.9033	23.7282	5
		MHAE	0.1490	0.0062	0.9161	18.0880	3
		MHE	0.1477	0.0062	0.9169	19.6782	4
		MOE	0.1987	0.0057	0.9278	16.4872	2

Note: ads, adsorption; des, desorption; GDW, generalized D’Arcy and Watt; MCPE, modified Chung-Pfost equation; MGAB, modified Guggenheim–Anderson–de Boer; MHAE, modified Halsey equation; MHE, modified Henderson equation; MOE, modified Oswin equation; *M*, equilibrium moisture content (%); *ERH*, equilibrium relative humidity (decimal); and *t*, temperature (°C). *RSS*, residue sum of squares; *SE*, standard error; *R*^2^, determination coefficient; and *MRE*, mean relative percentage error.

**Table 4:** Comparison of the statistical parameters of the equations for peony cake.

Type	Sorption	Eqn.	*RSS*	*SE*	*R* ^2^	*MRE* (%)	Order
M=f(ERH,t)	ads	GDW	34.9851	1.5211	0.9717	11.0034	6
		MCPE	130.7842	5.4493	0.8943	23.371	9
		MGAB	32.5339	1.3556	0.9737	9.2478	3
		MHAE	6.3095	0.2629	0.9949	4.482	1
		MHE	77.4203	3.2258	0.9374	21.0581	8
		MOE	24.3793	1.0158	0.9803	11.1334	7
		4-para	35.8598	1.5591	0.9711	10.9452	5
		Peleg	31.3199	1.3617	0.9747	10.6668	4
		Poly	10.3809	0.5191	0.9916	6.5192	2
	des	GDW	36.9296	1.6056	0.9696	10.7514	6
		MCPE	8.6716	0.3613	0.9929	15.626	9
		MGAB	42.2699	1.7612	0.9652	12.606	7
		MHAE	3.2661	0.1361	0.9973	4.1821	1
		MHE	47.2829	1.9701	0.9611	14.1362	8
		MOE	8.6716	0.3613	0.9929	5.3404	3
		4-para	35.4098	1.5396	0.9709	10.7514	5
		peleg	33.4292	1.4534	0.9725	10.1255	4
		poly	6.1221	0.3061	0.9949	4.2143	2
ERH=f(M,t)	ads	MCPE	8.46 × 10^−2^	3.53 × 10^−3^	0.9524	15.0804	4
		MGAB	7.51 × 10^−1^	3.13 × 10^−3^	0.9577	14.5715	3
		MHAE	2.31 × 10^−2^	9.65 × 10^−3^	0.9869	10.2108	1
		MHE	9.12 × 10^−2^	3.80 × 10^−3^	0.9487	17.3446	5
		MOE	4.16 × 10^−2^	1.73 × 10^−3^	0.9766	10.7351	2
	des	MCPE	4.38 × 10^−2^	1.82 × 10^−3^	0.9754	10.614	4
		MGAB	4.82 × 10^−2^	2.01 × 10^−3^	0.9729	10.4308	3
		MHAE	1.38 × 10^−2^	5.73 × 10^−4^	0.9923	8.249	2
		MHE	4.66 × 10^−2^	1.94 × 10^−3^	0.9738	12.1176	5
		MOE	1.46 × 10^−2^	6.10 × 10^−4^	0.9918	5.5025	1

Notes: ads, adsorption; des, desorption; GDW, generalized D’Arcy and Watt; MCPE, modified Chung-Pfost equation; MGAB, modified Guggenheim–Anderson–de Boer; MHAE, modified Halsey; MHE, modified Henderson equation; MOE, modified Oswin equation; *M*, equilibrium moisture content (%); *ERH*, equilibrium relative humidiy (decimal); and *t*, temperature (°C). *RSS*, residue sum of squares; *SE*, standard error; *R*^2^, determination coefficient; and *MRE*, mean relative percentage error.

**Table 5:** The adsorption polynomial parameters of peony samples.

Samples	Poly	Parameters						Statistical	Parameters	Residual Plot
	a	b	c	d	e	f	g	*R* ^2^	*MRE* (%)	
a1	45.573	−35.618	9.661	−0.164	0.0999	−0.221	8.752	0.9951	2.7942	R
a2	17.693	−15.118	6.006	0.186	−0.159	−0.154	9.283	0.9986	0.8326	R
a3	22.975	−20.728	7.533	0.268	−0.165	−0.198	9.939	0.9987	1.0545	R
a4	28.248	−26.412	9.371	0.223	−0.149	−0.202	10.036	0.9976	1.4824	R
a5	24.301	−20.114	6.723	0.144	−0.105	−0.188	9.689	0.9982	1.1809	R
a6	46.354	−35.921	9.274	−0.136	0.146	−0.271	9.941	0.9965	2.7735	R
a7	34.583	−25.205	7.422	−0.002933	0.002154	−0.255	9.935	0.9974	2.0931	R
a8	32.109	−28.267	10.789	0.179	−0.152	−0.231	9.687	0.9976	2.1047	R
a9	88.014	−86.802	29.926	−0.0426	−0.0483	−0.2407	8.208	0.9931	5.1622	R
a10	78.225	−68.235	19.854	−0.345	0.204	−0.242	8.731	0.9888	4.7410	R
Mean (seed)	41.834	−36.327	11.716	0.03283	−0.03452	−0.219	9.411	0.9968	2.2002	R
a11 (cake)	120.234	−104.084	28.725	−1.086	0.879	−0.323	7.583	0.9916	6.5192	R

Note: Mean (seed) is the mean of the EMC/ERH data of ten species of peony seeds; a, b, c, d, e, f, and g are the parameters of Poly; *R*^2^, determination coefficient; and *MRE*, mean relative percentage error. R shows that the residual plot is a random distribution.

**Table 6:** The desorption polynomial parameters of peony samples.

Samples	Poly	Parameters						Statistical	Parameters	Residual Plot
	a	b	c	d	e	f	g	*R* ^2^	*MRE* (%)	
a1	−22.511	5.083	19.765	1.303	−0.969	−0.393	15.177	0.9879	4.2938	R
a2	−25.842	16.588	2.423	0.611	−0.164	−0.397	16.028	0.9964	1.3567	R
a3	−28.513	9.996	12.807	1.156	−0.653	−0.378	15.307	0.9935	2.3458	R
a4	−27.365	9.856	13.125	1.153	−0.686	−0.378	15.468	0.9936	2.3525	R
a5	−29.516	15.304	9.148	0.969	−0.522	−0.386	15.627	0.9944	2.0952	R
a6	−6.143	−4.292	21.082	0.939	−0.767	−0.325	12.386	0.9927	3.3786	R
a7	−22.221	3.84	20.834	1.306	−0.971	−0.394	14.700	0.9929	3.7064	R
a8	−28.464	6.518	19.091	1.424	−0.956	−0.423	15.938	0.9934	3.1576	R
A9	−5.148	−14.809	34.827	1.634	−1.441	−1.343	13.828	0.9885	4.8112	R
a10	−23.601	13.591	21.778	1.337	−1.163	−0.335	14.395	0.9816	4.7194	R
Mean (seed)	−21.928	6.201	17.454	1.182	−0.828	−0.375	14.891	0.9924	2.9223	R
a11 (cake)	97.947	−92.631	37.901	−0.581	0.349	−0.238	5.936	0.9949	4.2143	R

Note: Mean (seed) is the mean of the EMC/ERH data of ten species of peony seeds; a, b, c, d, e, f, and g are the parameters of Poly; *R*^2^, determination coefficient; and *MRE*, mean relative percentage error. R shows that the residual plot is random distribution.

**Table 7:** The equation parameters and key statistical parameters of MHAE in the form of M=f(ERH,t).

Sorption	Samples	MHAE	Parameters		Statistical	Parameters	Residual Plot
		a	b	c	*R* ^2^	*MRE* (%)	
Adsorption	a1	4.371	−5.473 × 10^−2^	1.924	0.9704	9.1929	R
	a2	8.049	−7.875 × 10^−2^	3.423	0.9417	6.5179	R
	a3	6.273	−6.217 × 10^−2^	2.685	0.9539	7.6345	R
	a4	6.439	−6.534 × 10^−2^	2.706	0.9489	7.9956	R
	a5	6.601	−6.817 × 10^−2^	2.801	0.9509	7.5813	R
	a6	4.355	−5.129 × 10^−2^	1.875	0.9771	8.1736	R
	a7	4.719	−6.256 × 10^−2^	1.983	0.9705	9.0746	R
	a8	5.226	−6.684 × 10^−2^	2.175	0.9639	8.9608	R
	a9	3.728	−4.423 × 10^−2^	1.592	0.9714	11.5347	R
	a10	3.969	−4.499 × 10^−2^	1.699	0.9685	10.6413	R
	Mean (seed)	5.025	−5.689 × 10^−2^	2.141	0.9649	8.8038	R
	a11 (cake)	3.523	−3.306 × 10^−2^	1.493	0.9949	4.4820	R
Desorption	a1	9.502	−1.264 × 10^−1^	3.029	0.8731	15.9933	P
	a2	14.724	−1.471 × 10^−1^	5.156	0.7815	11.5991	P
	a3	11.526	−1.214 × 10^−1^	3.946	0.8143	13.8179	P
	a4	11.396	−1.239 × 10^−1^	3.849	0.8332	13.1151	P
	a5	12.258	−1.328 × 10^−1^	4.159	0.8136	13.1686	P
	a6	7.521	−9.390 × 10^−2^	2.542	0.9177	14.4335	P
	a7	9.241	−1.269 × 10^−1^	2.951	0.8722	17.9361	P
	a8	10.399	−1.355 × 10^−1^	3.331	0.8395	17.6709	P
	a9	7.309	−9.140 × 10^−2^	2.312	0.9138	17.2737	P
	a10	7.934	−9.316 × 10^−2^	2.539	0.8947	15.0419	P
	Mean (seed)	9.544	−1.141 × 10^−1^	3.141	0.8760	14.1612	P
	a11 (cake)	4.277	−3.710 × 10^−2^	1.686	0.9973	4.1821	R

Note: Mean (seed) is the mean of the EMC/ERH data of ten species of peony seeds; a, b, and c are the parameters of MHAE; *R*^2^, determination coefficient; and *MRE*, mean relative percentage error.

**Table 8:** The GDW parameters and isotherm types of peony samples.

Sorption	Samples	GDW	Parameters			Statistical	Parameters	Isotherm Type
		a	b	c	d	*R* ^2^	*MRE* (%)	
Adsorption	a1	3.554	9.694 × 10^12^	0.929	0.657	0.9239	14.6662	Type II
	a2	5.047	3.256 × 10^12^	0.899	0.316	0.8691	10.1161	Type II
	a3	4.631	−1.385 × 10^13^	0.892	0.478	0.9038	11.7885	Type III-like
	a4	4.839	1.141 × 10^13^	0.912	0.412	0.8967	11.9969	Type II
	a5	4.711	−9.485 × 10^12^	0.904	0.412	0.8929	11.5785	Type III-like
	a6	3.582	−1.296 × 10^13^	0.842	0.842	0.9334	14.9707	Type III-like
	a7	3.435	−3.018 × 10^13^	0.893	0.837	0.9059	17.2059	Type III-like
	a8	3.811	2.265 × 10^12^	0.898	0.667	0.8933	16.4211	Type II
	a9	3.792	−2.949 × 10^13^	0.959	0.738	0.9411	16.5627	Type III-like
	a10	4.008	−3.366 × 10^13^	0.956	0.663	0.9392	13.8851	Type III-like
	Mean (seed)	4.149	1.472 × 10^9^	0.9223	0.569	0.9189	13.6901	Type II
	a11 (cake)	3.683	−8.044 × 10^12^	0.931	1.226	0.9717	11.0034	Type III-like
Desorption	a1	3.704	1.413 × 10^12^	0.223	13.014	0.7498	22.6174	Type II
	a2	4.421	−4.561 × 10^12^	−0.885	−4.009	0.7044	13.4956	Type II like
	a3	4.091	−1.058 × 10^20^	−0.291	−11.769	0.7561	16.3858	Type II like
	a4	4.295	−5.299 × 10^20^	−0.154	−19.214	0.7531	16.6736	Type II like
	a5	4.138	−4.341 × 10^19^	−0.402	−8.648	0.7378	16.1379	Type II like
	a6	3.379	−5.429 × 10^11^	0.437	6.297	0.8431	19.9623	Type III-like
	a7	3.277	−2.390 × 10^10^	0.187	18.531	0.7574	24.0008	Type III-like
	a8	3.579	−2.001 × 10^10^	0.005705	617.527	0.7271	23.1458	Type III-like
	a9	3.835	−5.207 × 10^9^	0.556	4.658	0.832	25.9515	Type III-like
	a10	3.851	1.883 × 10^9^	0.407	7.583	0.8339	20.4856	Type II
	Mean (seed)	3.961	−2.788 × 10^7^	0.1771	15.985	0.7849	19.2136	Type III-like
	a11 (cake)	4.004	1.506 × 10^10^	0.865	1.612	0.9696	10.7514	Type II

Note: Mean (seed) is the mean of EMC/ERH data of ten species of peony seeds; a, b, c and d are the parameters of GDW; *R*^2^, determination coefficient; and *MRE*, mean relative percentage error.

**Table 9:** The monolayer moisture content estimated by MGAB for peony samples.

Sorption	Samples	MGAB	Parameters		Statistical	Parameter
		a	b	c	*R* ^2^	*MRE* (%)
Adsorption	a1	3.122	8.829 × 10^−1^	3.017 × 10^9^	0.9187	15.0546
	a2	4.328	6.783 × 10^−1^	4.120 × 10^9^	0.8266	11.4414
	a3	4.009	7.724 × 10^−1^	4.531 × 10^9^	0.8868	12.5197
	a4	4.087	7.706 × 10^−1^	9.542 × 10^9^	0.8711	12.9304
	a5	4.011	7.568 × 10^−1^	5.372 × 10^9^	0.8672	12.5898
	a6	3.385	8.888 × 10^−1^	6.245 × 10^8^	0.9326	15.0996
	a7	3.251	8.718 × 10^−1^	1.291 × 10^9^	0.9051	17.3294
	a8	3.419	8.447 × 10^−1^	2.418 × 10^9^	0.8886	16.7822
	a9	3.371	9.355 × 10^−1^	9.711 × 10^8^	0.9386	16.9557
	a10	3.463	9.189 × 10^−1^	4.962 × 10^9^	0.9343	14.2796
	Mean (seed)	3.567	8.512 × 10^−1^	2.029 × 10^9^	0.9089	14.3511
	a11 (cake)	4.281	9.358 × 10^−1^	7.426 × 10^2^	0.9737	9.2478
Desorption	a1	5.869	7.197 × 10^−1^	−1.381 × 10^10^	0.7059	26.836
	a2	6.815	5.247 × 10^−1^	−3.906 × 10^10^	0.6212	16.0858
	a3	6.551	6.209 × 10^−1^	−1.565 × 10^10^	0.6883	19.8533
	a4	6.548	6.303 × 10^−1^	−2.029 × 10^10^	0.6951	19.8468
	a5	6.605	6.014 × 10^−1^	−2.191 × 10^10^	0.6665	19.3406
	a6	5.394	7.794 × 10^−1^	−6.292 × 10^9^	0.8061	24.5650
	a7	5.655	7.288 × 10^−1^	−1.183 × 10^10^	0.7063	29.2203
	a8	6.059	6.858 × 10^−1^	−2.436 × 10^10^	0.6688	27.9902
	a9	6.079	8.137 × 10^−1^	−8.412 × 10^9^	0.8013	30.0436
	a10	6.519	7.787 × 10^−1^	−8.975 × 10^9^	0.7902	25.0924
	Mean (seed)	6.147	7.046 × 10^−1^	−9.824 × 10^9^	0.7387	23.0729
	a11 (cake)	4.833	9.136 × 10^−1^	−8.259 × 10^9^	0.9652	12.6060

Note: Mean (seed) is the mean of the EMC/ERH data of ten species of peony seeds; a, b, and c are the parameters of MGAB; *R*^2^, determination coefficient; and *MRE*, mean relative percentage error.

**Table 10:** The absolutely safe storage MC of peony samples.

Samples	ERH = 60%			ERH = 65%		
	20 °C	25 °C	30 °C	20 °C	25 °C	30 °C
			MC	(% w.b.)		
a1	13.90	11.37	8.84	14.54	12.18	9.82
a2	12.36	10.99	9.61	12.61	11.38	10.15
a3	13.36	11.59	9.82	13.74	12.17	10.60
a4	13.49	11.62	9.74	13.91	12.23	10.55
a5	13.24	11.49	9.74	13.62	12.04	10.46
a6	13.22	10.98	8.75	14.05	11.92	9.78
a7	13.65	11.12	8.59	14.29	11.93	9.56
a8	13.91	11.49	9.07	14.43	12.22	10.00
a9	15.89	12.80	9.70	17.01	14.06	11.12
a10	16.23	13.47	10.71	17.29	14.66	12.03
Mean (seed)	13.93	11.69	9.46	14.55	12.48	10.41
a11 (cake)	11.73	10.54	9.35	13.20	11.92	10.64

Note: Mean (seed) is the mean of EMC/ERH data of ten species of peony seeds.

**Table 11:** The MCPE and MHAE of peony seed and cake samples in the form of ERH=f(M,t).

Equation	Sorption	Samples	Equation	Parameters		Statistical	Parameter
			a	b	c	*R* ^2^	*MRE* (%)
MCPE	ads	Seed	99.529	−12.278	0.377	0.8903	30.0844
		Cake	100.895	0.707	0.208	0.9524	15.0804
	des	Seed	69.159	−14.522	0.238	0.9714	9.3477
		Cake	101.115	1.549	0.176	0.9754	10.614
MHAE	ads	Seed	6.119	−0.1089	2.598	0.9252	24.8929
		Cake	4.136	−0.046	1.625	0.9869	10.2108
	des	Seed	7.405	−0.1164	2.188	0.9325	16.2903
		Cake	4.276	−0.0432	1.615	0.9923	8.2490
MOE	ads	Seed	13.001	−0.2601	3.456	0.9036	28.4658
		Cake	13.396	−0.2089	2.294	0.9766	10.7351
	des	Seed	21.301	−0.4639	3.184	0.9436	15.3589
		Cake	15.318	−0.2347	2.308	0.9918	5.5025

Note: For peony seed samples, the average EMC/ERH of ten species of seed samples was used. The peony cake is the a11 sample. MCPE, modified Chung-Pfost equation; MHAE, modified Halsey equation; MOE, modified Oswin equation; a, b, and c are the equation parameters; *R*^2^, determination coefficient; and *MRE*, mean relative percentage error.

## Data Availability

The original contributions presented in this study are included in this article/Supplementary Materials; further inquiries can be directed to the corresponding author.

## Supplementary Materials

The following supporting information can be downloaded at: https://www.mdpi.com/article/10.3390/foods15081298/s1, Figure S1: The plot of EMC residual of the EMC/ERH data of average ten varieties of peony seeds and one peony cake fitted by Poly equation; Figure S2: The plot of EMC residual of the EMC/ERH data of average ten varieties of peony seeds and one peony cake fitted by MHAE.
