# Supplementary material for: Seven-Parameter Polynomial Fits Better to the Moisture Sorption Isotherms of Oil-Type Peony Seeds and Cake
Source: Foods. 2026 Apr 9;15(8):1298. doi: 10.3390/foods15081298 (PMC13114299; doi:10.3390/foods15081298)
Supplement: Supplementary file 1 [file foods-15-01298-s001.zip › foods-4043921-supplementary.pdf]

Supplement

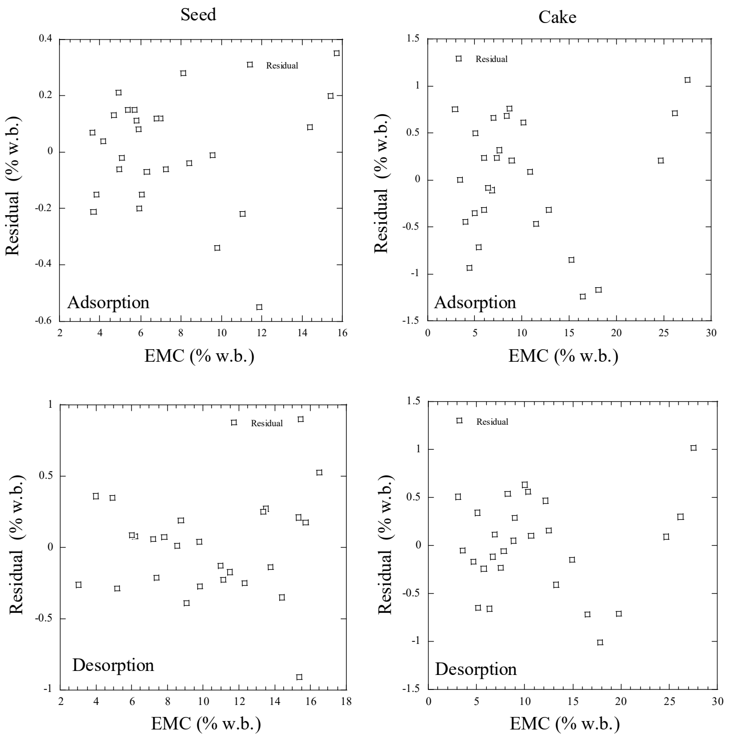

Figure S1. The plot of EMC residual of the EMC/ERH data of average ten varieties of peony seeds and one peony cake fitted by Poly equation.

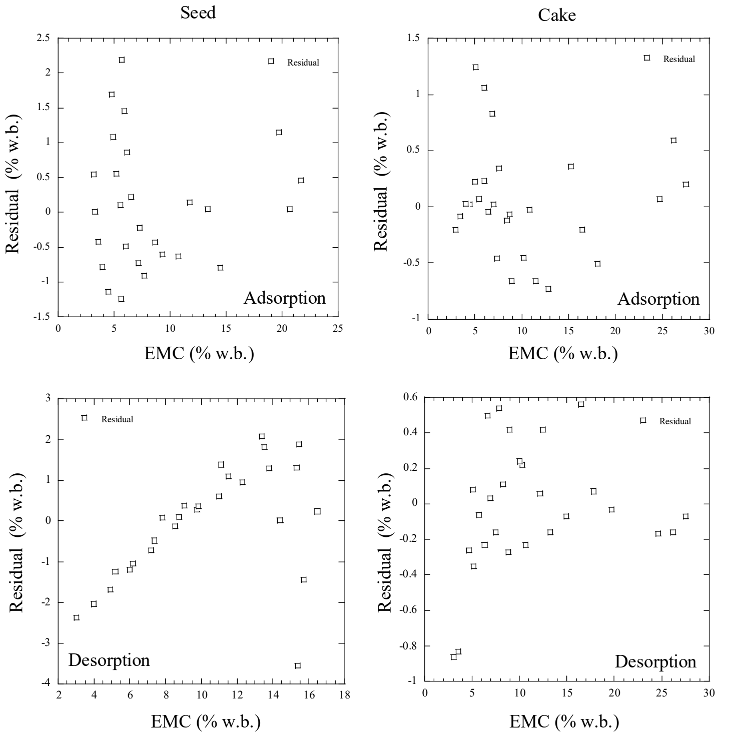

Figure S2. The plot of EMC residual of the EMC/ERH data of average ten varieties of peony seeds and one peony cake fitted by MHAE.
